# Perineural Invasion Is a Strong Prognostic Factor but Not a Predictive Factor of Response to Adjuvant Chemotherapy in Node-Negative Colon Cancer

**DOI:** 10.3389/fonc.2021.663154

**Published:** 2021-03-30

**Authors:** Junhao Tu, Zongxi Yao, Wenqing Wu, Jianxiang Ju, Yinkai Xu, Yulin Liu

**Affiliations:** ^1^ Department of General Surgery, Suzhou Wuzhong People’s Hospital, Suzhou, China; ^2^ Department of General Surgery, The First Affiliated Hospital of Soochow University, Suzhou, China

**Keywords:** perineural invasion, prognostic, adjuvant chemotherapy, node-negative, colon cancer

## Abstract

**Purpose:**

To validate the prognostic value and evaluate the predictive value of response to adjuvant chemotherapy of perineural invasion (PNI) in node-negative colon cancer using the National Cancer Institute’s Surveillance, Epidemiology, and End Results (SEER) 18 tumor registry database.

**Methods:**

Patients diagnosed with colon cancer from the SEER database between January 1, 2010 and December 31, 2015 were identified. Chi-square analysis was performed to evaluate different demographic and clinical features of patients between PNI-negative (PNI (−)) and PNI-positive (PNI (+)) groups. Univariate and multivariate Cox proportional hazard regression models were built to examine the relationship of demographic and clinical features and survival outcomes with the hazard ratios (HRs) and 95% confidence intervals (CIs).

**Results:**

In total, 57,255 node-negative colon cancer patients were extracted from the SEER database. The receipt of chemotherapy was not an independent prognostic factor for CSS in T3 colon cancer with or without the presence of PNI (P >0.05). The receipt of chemotherapy was independently associated with 34.0% decreased risk of cancer-specific mortality compared with those without the receipt of chemotherapy in T4 colon cancer without the presence of PNI (HR = 0.660, 95%CI = 0.559–0.779, P <0.001); the receipt of chemotherapy was independently associated with 36.0% decreased risk of cancer-specific mortality compared with those without the receipt of chemotherapy in T4 colon cancer with the presence of PNI (HR = 0.640, 95%CI = 0.438–0.935, P = 0.021).

**Conclusions:**

The present study demonstrated the poor prognosis of PNI (+) in both stage I and II colon cancer. However, the presence of PNI was not a predictive factor of response to adjuvant chemotherapy in node-negative colon cancer.

## Introduction

As one of the most commonly diagnosed malignant tumors, colon cancer is an important public health issue worldwide (1). Currently, the current standards for clinical treatment and prognostic prediction of survival and recurrence in colon cancer are principally based on pathological staging of the American Joint Committee on Cancer (AJCC) Tumor-Node-Metastasis (TNM) staging system. According to the clinical guidelines of National Comprehensive Cancer Network, stage III colon cancer deserve adjuvant chemotherapy for better prognosis (2–8). However, the TNM staging is not accurate enough to stratify those node-negative (stage I/II) colon cancer patients, and previous studies have indicated the prognostic implications of various histopathological factors (9–12).

In addition to direct growth, tumor cells can disseminate through the blood and lymph channels or grow along the nerves. Positive perineural invasion (PNI) is therefore defined as the invasion spreading in or around the neural tissue and/or spread along nerve sheaths, even in the absence of lymphovascular invasion (LVI) or lymph node metastasis (12–16). PNI would finally occur after changes in nerve cells and supporting cells, changes and metastasis of the perineural matrix, injury and regeneration of nerves; adhesion of nerve cells and tumor cells; and escape, autophagy and apoptosis of tumor cells and so on (17). It has been widely reported that the presence of PNI would indicate more aggressive clinicopathological features, resulting in poor prognosis in colorectal cancer, and some previous studies found that PNI could be an indicator for the receipt of chemotherapy in colon cancer (12, 18–22). The prognostic value of PNI in colorectal cancer has been widely recognized, however, its predictive role for the receipt of adjuvant chemotherapy is less clear (23–25). Therefore, the present large population-based study was to validate the prognostic value and evaluate the predictive value of response to adjuvant chemotherapy of PNI in node-negative colon cancer.

## Materials and Methods

### Patients

Data used in the present study were retrieved from the National Cancer Institute’s Surveillance, Epidemiology, and End Results (SEER) 18 tumor registry database. The SEER database, which emphasized quality control and stipulates a less than five percent error rate, contained approximately 28% of the US population and included population demographic information, clinicopathological characteristics, treatment and survival information from more than three million patients (26). Using SEER*Stat version 8.3.6, patients diagnosed with colon cancer between January 1, 2010 and December 31, 2015 were identified. Personal information of patients was not involved in the present study, therefore, the requirement for informed consent was waived.

The patient characteristics extracted from the SEER database included T stage, age at diagnosis, race, sex, year of diagnosis, tumor grade, histological type, total number of lymph nodes examined, the receipt of chemotherapy and perineural invasion status. The exclusion criteria were (1) lack of positive histological confirmation, (2) race was unknown, (3) non-adenocarcinoma histologies, (4) not active follow-up, and (5) lack of radical surgery. In addition, only those patients without lymph node or distant metastasis and with known perineural invasion status were included into our analyses.

### Statistical Analysis

In the present study, Cancer-specific survival (CSS) was used as the survival endpoint and analyzed using the Kaplan–Meier method with log-rank test to evaluate the outcomes of different groups. Kaplan–Meier curves were often used to visually summarize time-to-event data, in which y axis indicated the proportion of individuals under risk of an event, and the x axis indicated time. The curves were often presented with 95% confidence intervals and a difference between curves can be tested statistically, most commonly using the log rank test (27). CSS was defined as the time between the diagnosis of colon cancer and cancer-specific death or the last follow up, mortality cases resulted from other causes were censored.

Chi-square analysis was performed to evaluate different demographic and clinical features of patients between PNI-negative (PNI (−)) and PNI-positive (PNI (+)) groups. The chi-square test commonly either compared the distribution of a categorical variable to a hypothetical distribution or tested whether the two categorical variables were independent. In our analyses, the chi-square test was used to evaluate the null hypothesis that two categorical variables (e.g., treatment group [male versus female] and outcome [PNI (−) versus PNI (+)]) were not associated with each other (28, 29).

Univariate and multivariate Cox proportional hazard regression models were built to examine the relationship of demographic and clinical features and survival outcomes with the hazard ratios (HRs) and 95% confidence intervals (CIs). The Cox proportional hazard regression model was to determine the extent to which changed in the risk factors affect the survival of colon cancer. The HR of each demographic or clinical feature in the model can be estimated according to the minimum, maximum, or standard deviation of the values of the demographic and clinical features (30). P values less than 0.05 were considered statistically significant. Statistical analyses were performed using the Statistical Product and Service Solutions (SPSS) Statics software (version 23; IBM Corporation, NY, USA).

## Results

### Patient Characteristics

In total, 57,255 node-negative colon cancer patients satisfying the inclusion and exclusion criteria were extracted from the SEER database. Among the whole cohort, 25,450 (44.5%) patients were diagnosed with stage I disease, 21,090 (26.8%) patients aged less than 65 years old, 28,369 (49.5%) patients were male, 2,372 (4.1%) patients were diagnosed with the presence of PNI. The median follow-up time for patients alive at last follow-up time was 37 months.

Different demographic and clinical features of patients between PNI (–) and PNI (+) groups were compared in **Table 1**. It was found that high T stage (4.4% VS. 24.7% for stage T1, 9.2% VS. 21.1% for stage T2, 62.4% VS. 46.4% for stage T3, 23.9% VS. 7.8% for stage T4, P <0.001), later year of diagnosis (13.7% VS. 16.4% for 2010, 15.4% VS. 16.7% for 2011, 17.5% VS. 16.9% for 2012, 18.0% VS. 16.6% for 2013, 17.5% VS. 16.7% for 2014, 17.8% VS. 16.7% for 2015, P = 0.003); higher tumor grade (4.9% VS. 10.9% for grade I, 68.7% VS. 73.0% for grade II, 21.3% VS. 10.1% for grade III, 3.7% VS. 2.1% for grade IV, P <0.001); adenocarcinoma (93.3% VS. 92.2% for adenocarcinoma, 6.7% VS. 7.8% for mucinous/signet-ring cell carcinoma, P = 0.042) and more lymph nodes examined (10.9% VS. 19.1% for less than 12 lymph nodes examined, 89.1% VS. 80.9% for more than 12 lymph nodes examined, P <0.001) were more likely to be associated with the presence of PNI. In addition, the presence of PNI was more likely to correlate with the receipt of chemotherapy (P <0.001). The associations of age at diagnosis, race and sex between PNI (−) and PNI (+) groups did not reach statistical significance (P >0.05).

**Table 1:** Demographic and clinical features of the patients according to perineural invasion status.

Feartue	No. of Patients (%)		*P*
	PNI (−) (N = 54,883)	PNI (+) (N = 2,372)	
**T stage**			<0.001
** T1**	13,552 (24.7)	104 (4.4)	
** T2**	11,575 (21.1)	219 (9.2)	
** T3**	25,459 (46.4)	1,481 (62.4)	
** T4**	4,297 (7.8)	568 (23.9)	
**Age at diagnosis**			0.672
**≤65**	20,226 (36.9)	864 (36.4)	
** >65**	34,657 (63.1)	1,508 (63.6)	
**Race**			0.199
**White**	44,381 (80.9)	1,895 (79.9)	
**Black**	6,219 (11.3)	297 (12.5)	
** Other**	4283 (7.8)	180 (7.6)	
**Sex**			0.418
** Male**	27,213 (49.6)	1,156 (48.7)	
** Female**	27,670 (50.4)	1,216 (51.3)	
**Year**			0.003
**2010**	8,980 (16.4)	326 (13.7)	
**2011**	9,185 (16.7)	366 (15.4)	
** 2012**	9,267 (16.9)	415 (17.5)	
** 2013**	9,107 (16.6)	428 (18.0)	
** 2014**	9,192 (16.7)	415 (17.5)	
** 2015**	9,152 (16.7)	422 (17.8)	
**Grade**			<0.001
** I**	6,003 (10.9)	116 (4.9)	
** II**	40,089 (73.0)	1,630 (68.7)	
** III**	5,567 (10.1)	505 (21.3)	
** IV**	1,156 (2.1)	88 (3.7)	
** Unknown**	2,068 (3.8)	33 (1.4)	
**Histological type**			0.042
** Adenocarcinoma**	50,600 (92.2)	2,214 (93.3)	
** Mucinous/signet-ring cell carcinoma**	4,282 (7.8)	158 (6.7)	
**Total number of lymph nodes examined**			<0.001
** <12**	10,487 (19.1)	259 (10.9)	
**≥12**	44,396 (80.9)	2,113 (89.1)	
**Chemotherapy**			<0.001
** No**	50,305 (91.7)	1,886 (79.5)	
** Yes**	4,578 (8.3)	486 (20.5)	

### Prognostic Significance of PNI in Node-Negative Colon Cancer

**Figure 1:**
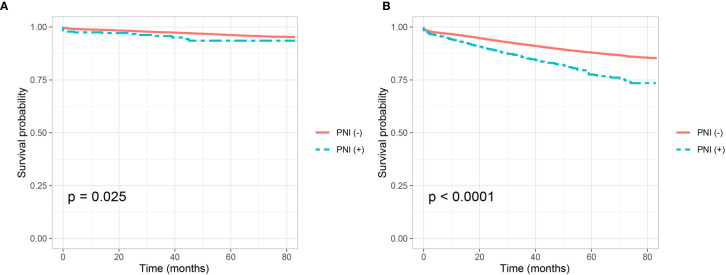
Kaplan–Meier curves for cancer-specific survival according to the perineural invasion status (PNI (−) VS. PNI (+) in **(A)** stage I colon cancer (P = 0.025) and **(B)** stage II colon cancer (P <0.001).

Shown as **Figures 1A, B**, we plotted the Kaplan–Meier CSS curves of node-negative colon cancer patients with the presence of PNI compared to those without the presence of PNI. Kaplan–Meier analyses showed that PNI (+) patents (5-year CSS rate = 93.6%) were significantly associated with poorer CSS compared with PNI (−) patents (5-year CSS rate = 96.2%) in stage I colon cancer (P = 0.025, **Figure 1A**). It was also found that PNI (+) patents (5-year CSS rate = 77.5%) were significantly associated with poorer CSS compared with PNI (−) patents (5-year CSS rate = 87.9%) in stage II colon cancer and the survival difference was widened in stage II colon cancer than in stage I colon cancer (P <0.0001, **Figure 1B**).

In addition, Cox proportional hazard regression models were completed to assess the independent prognostic factors for CSS in node-negative colon cancer, including T stage, age at diagnosis, race, sex, year of diagnosis, tumor grade, histological type, total number of lymph nodes examined and perineural invasion status (**Tables 2** **,** **3**). Only the predictors with P values less than 0.20 in the univariate analysis were entered into a multivariate Cox model. Multivariate survival analyses showed that T stage (P <0.001), age at diagnosis (P <0.001), race (P <0.001), sex (P <0.028) and total number of lymph nodes examined (P <0.001) were independent prognostic factors in stage I colon cancer (**Table 2**). After adjusting for other prognostic factors, more importantly, it was found that PNI (+) patients were independently associated 59.0% increased risk of colon cancer-specific mortality compared with PNI (−) patients in stage I colon cancer though the P value did not reach statistical significance, which might result from the small sample size (n = 323) of stage I colon cancer patients with the presence of PNI (HR = 1.590, 95%CI = 0.951–2.658, P = 0.077, using no PNI as the reference; **Table 2**).

**Table 2:** Univariate and multivariate survival analyses of stage I colon cancer.

Variable	Univariate		Multivariate	
	HR (95%CI)	*P*	HR (95%CI)	*P*
**T stage**		<0.001		<0.001
** T1**	Reference		Reference	
** T2**	1.550 (1.336–1.798)		1.552 (1.324–1.819)	
**Age at diagnosis**		<0.001		<0.001
** ≤65**	Reference		Reference	
**>65**	2.276 (1.913–2.707)		2.281 (1.913–2.720)	
**Race**		<0.001		<0.001
**White**	Reference		Reference	
**Black**	1.493 (1.224–1.822)	<0.001	1.703 (1.393–2.081)	<0.001
**Other**	0.804 (0.589–1.097)	0.168	0.874 (0.641–1.193)	0.397
**Sex**		0.151		0.028
**Male**	Reference		Reference	
** Female**	0.897 (0.774–1.040)		0.847 (0.730–0.982)	
**Year**		0.106		0.122
**2010**	Reference		Reference	
** 2011**	0.929 (0.745–1.160)	0.517	0.929 (9.744–1.160)	0.516
** 2012**	0.900 (0.709–1.143)	0.388	0.912 (0.718–1.159)	0.451
** 2013**	1.195 (0.941–1.517)	0.143	1.223 (0.963–1.553)	0.100
** 2014**	0.885 (0.669–1.172)	0.394	0.921 (0.695–1.219)	0.564
** 2015**	0.808 (0.582–1.120)	0.200	0.852 (0.614–1.182)	0.337
**Grade**		0.009		0.085
** I**	Reference		Reference	
** II**	1.444 (1.146–1.820)	0.002	1.360 (1.076–1.718)	0.010
** III**	1.583 (1.117–2.243)	0.010	1.459 (1.026–2.075)	0.035
** IV**	1.413 (0.684–2.919)	0.351	1.319 (0.637–2.730)	0.456
** Unknown**	1.041 (0.704–1.540)	0.839	1.085 (0.732–1.610)	0.685
**Histological type**		0.999		
** Adenocarcinoma**	Reference			
** Mucinous/signet-ring cell carcinoma**	1.000 (0.694–1.442)			
**Total number of lymph nodes examined**		<0.001		<0.001
** <12**	Reference		Reference	
** ≥12**	0.726 (0.622–0.848)		0.614 (0.521–0.722)	
**Perineural invasion**		0.028		0.077
** None**	Reference		Reference	
** Present**	1.777 (1.066–2.964)		1.590 (0.951–2.658)	

**Table 3:** Univariate and multivariate survival analyses of stage II colon cancer.

Variable	Univariate		Multivariate	
	HR (95%CI)	*P*	HR (95%CI)	*P*
**T stage**		<0.001		<0.001
** T3**	Reference		Reference	
** T4**	2.847 (2.631–3.079)		2.806 (2.591–3.039)	
**Age at diagnosis**		<0.001		<0.001
** ≤65**	Reference		Reference	
**>65**	1.833 (1.683–1.995)		1.894 (1.738–2.064)	
**Race**		<0.001		<0.001
**White**	Reference		Reference	
**Black**	1.146 (1.027–1.279)	0.015	1.268 (1.135–1.417)	<0.001
** Other**	0.785 (0.673–0.915)	0.002	0.825 (0.707–0.962)	0.014
**Sex**		0.005		0.149
** Male**	Reference		Reference	
** Female**	1.111 (1.033–1.196)		1.056 (0.981–1.136)	
**Year**		0.136		0.426
**2010**	Reference		Reference	
** 2011**	0.994 (0.980–1.109)	0.909	0.993 (0.890–1.108)	0.901
** 2012**	0.957 (0.853–1.073)	0.449	0.961 (0.857–1.078)	0.499
** 2013**	0.926 (0.818–1.048)	0.223	0.947 (0.836–1.072)	0.386
** 2014**	0.883 (0.771–1.012)	0.074	0.917 (0.800–1.051)	0.212
** 2015**	0.824 (0.704–0.966)	0.017	0.855 (0.729–1.002)	0.053
**Grade**		<0.001		0.020
** I**	Reference		Reference	
** II**	1.018 (0.877–1.183)	0.810	1.023 (0.881–1.188)	0.764
** III**	1.356 (1.147–1.603)	<0.001	1.203 (1.016–1.424)	0.032
** IV**	1.244 (0.974–1.590)	0.080	1.110 (0.868–1.419)	0.404
** Unknown**	1.313 (0.954–1.807)	0.095	1.169 (0.849–1.610)	0.337
**Histological type**		0.682		
** Adenocarcinoma**	Reference			
** Mucinous/signet-ring cell carcinoma**	1.025 (0.911–1.154)			
**Total number of lymph nodes examined**		<0.001		<0.001
** <12**	Reference		Reference	
** ≥12**	0.525 (0.479–0.576)		0.541 (0.493–0.594)	
**Perineural invasion**		<0.001		<0.001
** None**	Reference		Reference	
** Present**	1.841 (1.636–2.072)		1.607 (1.426–1.812)	

In **Table 3**, multivariate survival analyses showed that T stage (P <0.001), age at diagnosis (P <0.001), race (P <0.001), tumor grade (P = 0.020) and total number of lymph nodes examined (P <0.001) were independent prognostic factors in stage II colon cancer. After adjusting for other prognostic factors, more importantly, it was found that PNI (+) patients were independently associated 60.7% increased risk of colon cancer-specific mortality compared with PNI (−) patients in stage II colon cancer (HR = 1.607, 95%CI = 1.426–1.812, P <0.001, using no PNI as the reference; **Table 3**).

### PNI Is Not a Predictive Factor of Response to Adjuvant Chemotherapy in Stage II Colon Cancer

**Figure 2:**
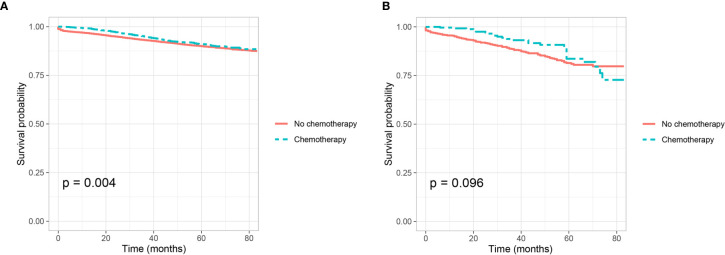
Kaplan–Meier curves for cancer-specific survival according to the receipt of chemotherapy (no chemotherapy VS. chemotherapy) in stage IIA colon cancer **(A)** with the presence of perineural invasion (P = 0.004) and **(B)** without the presence of perineural invasion (P = 0.096).

Adjuvant chemotherapy was not traditionally used in stage I colon cancer, we then evaluate whether PNI is a predictive factor of response to adjuvant chemotherapy in stage II colon cancer. Shown as **Figures 2A, B**, we plotted the Kaplan–Meier CSS curves of T3 colon cancer patients with the receipt of chemotherapy compared to those without the receipt of chemotherapy. Kaplan–Meier analyses showed that patents with the receipt of chemotherapy (5-year CSS rate = 91.1%) were significantly associated with better CSS compared to those without the receipt of chemotherapy (5-year CSS rate = 90.0%) in T3 colon cancer without the presence of PNI (P = 0.004, **Figure 2A**). It was also found that the receipt of chemotherapy (5-year CSS rate = 83.6%) was associated with better CSS compared with those without the receipt of chemotherapy (5-year CSS rate = 81.4%) in T3 colon cancer with the presence of PNI, but the P value did not reach statistical significance (P = 0.096, **Figure 2B**).

Cox proportional hazard regression models were completed to assess the independent prognostic factors for CSS in T3 colon cancer (**Table 4** and **Table S1**). After adjusting for other prognostic factors, it was found that the receipt of chemotherapy was not an independent prognostic factor for CSS in T3 colon cancer without the presence of PNI (HR = 0.943, 95%CI = 0.802–1.108, P = 0.473, using no PNI and no chemotherapy as the reference; **Table 4**); the receipt of chemotherapy was not an independent prognostic factor for CSS in T3 colon cancer with the presence of PNI (HR = 0.927, 95%CI = 0.613–1.400, P = 0.717, using the presence of PNI and no chemotherapy as the reference; **Table S1**).

**Table 4:** Univariate and multivariate survival analyses of T3N0M0 colon cancer.

Variable	Univariate		Multivariate	
	HR (95%CI)	*P*	HR (95%CI)	*P*
**Age at diagnosis**		<0.001		<0.001
** ≤65**	Reference		Reference	
** >65**	2.072 (1.863–2.306)		2.038 (1.825–2.276)	
**Race**		0.001		<0.001
** White**	Reference		Reference	
** Black**	1.138 (0.997–1.299)	0.056	1.260 (1.102–1.440)	0.001
** Other**	0.744 (0.616–0.899)	0.002	0.786 (0.650–0.950)	0.013
**Sex**		0.292		
** Male**	Reference			
** Female**	1.048 (0.960–1.145)			
**Year**		0.068		0.204
** 2010**	Reference		Reference	
** 2011**	0.957 (0.840–1.091)	0.512	0.965 (0.847–1.099)	0.591
** 2012**	0.946 (0.825–1.084)	0.424	0.958 (0.836–1.099)	0.539
** 2013**	0.871 (0.750–1.012)	0.072	0.878 (0.755–1.020)	0.089
** 2014**	0.851 (0.721–1.003)	0.055	0.892 (0.756–1.052)	0.175
** 2015**	0.758 (0.623–0.922)	0.006	0.794 (0.652–0.966)	0.021
**Grade**		0.192		0.406
** I**	Reference		Reference	
** II**	1.050 (0.878–1.254)	0.595	1.057 (0.885–1.263)	0.542
** III**	1.210 (0.985–1.485)	0.069	1.186 (0.965–1.457)	0.105
** IV**	1.063 (0.773–1.462)	0.708	1.058 (0.768–1.455)	0.731
** Unknown**	1.220 (0.812–1.834)	0.339	1.139 (0.758–1.713)	0.531
**Histological type**		0.459		
** Adenocarcinoma**	Reference			
** Mucinous/signet-ring cell carcinoma**	0.944 (0.811–1.099)			
**Total number of lymph nodes examined**		<0.001		<0.001
** <12**	Reference		Reference	
** ≥12**	0.539 (0.482–0.604)		0.561 (0.501–0.629)	
**Perineural invasion, chemotherapy**		<0.001		<0.001
** None, no/unknown**	Reference		Reference	
** None, yes**	0.793 (0.678–0.928)	0.004	0.943 (0.802–1.108)	0.473
** Present, no/unknown**	1.749 (1.476–2.073)	<0.001	1.761 (1.485–2.088)	<0.001
** Present, yes**	1.269 (0.868–1.856)	0.219	1.632 (1.113–2.391)	0.012

**Figure 3:**
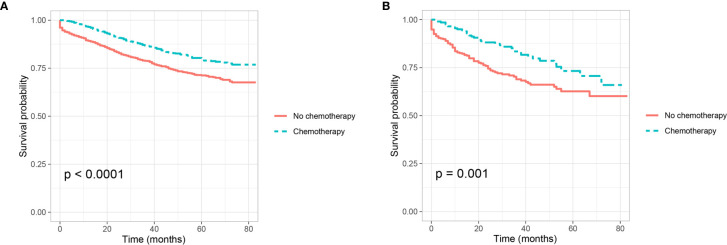
Kaplan–Meier curves for cancer-specific survival according to the receipt of chemotherapy (no chemotherapy VS. chemotherapy) in stage IIB colon cancer **(A)** with the presence of perineural invasion (P <0.001) and **(B)** without the presence of perineural invasion (P = 0.001).

Shown as **Figures 3A, B**, we plotted the Kaplan–Meier CSS curves of T4 colon cancer patients with the receipt of chemotherapy compared to those without the receipt of chemotherapy. Kaplan–Meier analyses showed that patents with the receipt of chemotherapy (5-year CSS rate = 80.1%) were significantly associated with better CSS compared to those without the receipt of chemotherapy (5-year CSS rate = 71.2%) in T4 colon cancer without the presence of PNI (P <0.0001, **Figure 3A**). It was also found that the receipt of chemotherapy (5-year CSS rate = 73.3%) was significantly associated with better CSS compared with those without the receipt of chemotherapy (5-year CSS rate = 62.7%) in T4 colon cancer with the presence of PNI (P = 0.001, **Figure 3B**).

In addition, Cox proportional hazard regression models were completed to assess the independent prognostic factors for CSS in T4 colon cancer **Table 5** and **Table S2**). After adjusting for other prognostic factors, it was found that the receipt of chemotherapy was independently associated with 34.0% decreased risk of cancer-specific mortality compared with those without the receipt of chemotherapy in T4 colon cancer without the presence of PNI (HR = 0.660, 95%CI = 0.559–0.779, P <0.001, using no PNI and no chemotherapy as the reference; **Table 5**); the receipt of chemotherapy was independently associated with 36.0% decreased risk of cancer-specific mortality compared with those without the receipt of chemotherapy in T4 colon cancer with the presence of PNI (HR = 0.640, 95%CI = 0.438–0.935, P =0.021, using the presence of PNI and no chemotherapy as the reference; **Table S2**).

**Table 5:** Univariate and multivariate survival analyses of T4N0M0 colon cancer.

Variable	Univariate		Multivariate	
	HR (95%CI)	*P*	HR (95%CI)	*P*
**Age at diagnosis**		<0.001		<0.001
** ≤65**	Reference		Reference	
** >65**	1.655 (1.437–1.908)		1.417 (1.216–1.651)	
**Race**		0.153		0.014
**White**	Reference		Reference	
**Black**	1.183 (0.971–1.440)	0.095	1.324 (1.085–1.616)	0.006
** Other**	0.897 (0.689–1.167)	0.419	0.913 (0.701–1.189)	0.500
**Sex**		0.003		0.051
** Male**	Reference		Reference	
**Female**	1.223 (1.072–1.395)		1.141 (0.999–1.303)	
**Year**		0.690		
**2010**	Reference			
** 2011**	1.068 (0.873–1.308)	0.522		
** 2012**	0.946 (0.765–1.169)	0.606		
** 2013**	1.048 (0.841–1.307)	0.676		
** 2014**	0.909 (0.715–1.157)	0.439		
** 2015**	0.927 (0.707–1.216)	0.483		
**Grade**		0.001		0.016
** I**	Reference		Reference	
** II**	0.928 (0.706–1.221)	0.595	0.950 (0.722–1.252)	0.717
** III**	1.289 (0.960–1.730)	0.092	1.246 (0.925–1.678)	0.147
** IV**	1.170 (0.788–1.736)	0.436	1.168 (0.786–1.736)	0.442
** Unknown**	1.097 (0.652–1.847)	0.726	1.183 (0.702–1.993)	0.528
**Histological type**		0.508		
** Adenocarcinoma**	Reference			
** Mucinous/signet-ring cell carcinoma**	0.938 (0.775–1.134)			
**Total number of lymph nodes examined**		<0.001		<0.001
** <12**	Reference		Reference	
** ≥12**	0.521 (0.443–0.612)		0.508 (0.431–0.598)	
**Perineural invasion, chemotherapy**		<0.001		<0.001
** None, no/unknown**	Reference		Reference	
** None, yes**	0.583 (0.499–0.683)	<0.001	0.660 (0.559–0.779)	<0.001
** Present, no/unknown**	0.802 (0.577–1.114)	0.188	1.458 (1.174–1.811)	0.001
** Present, yes**	1.497 (1.207–1.855)	<0.001	0.933 (0.668–1.33)	0.684

## Discussion

PNI was reported in head and neck cancers by Russian and French researchers since the 1800s (12). Subsequently, the prognostic value was then reported by Bataskis until the 1970s, described as “tumor invasion in, around, and through the nerves” (31). PNI would finally occur after changes in nerve cells and supporting cells, changes and metastasis of the perineural matrix, injury and regeneration of nerves; adhesion of nerve cells and tumor cells; and escape, autophagy and apoptosis of tumor cells and so on (17). In addition, slug was reported to promote PNI and distant metastasis of tumor cells through the MAPK signal pathway (32, 33). And the expression of the L1 cell adhesion molecule could promote the occurrence of PNI by influencing the migration of nerve cells (34).

Some studies have reported that the presence of perineural invasion would indicate more aggressive clinicopathological features (35). In this study, we have showed that the presence of PNI was significantly correlated with high T stage, later year of diagnosis, higher tumor grade, adenocarcinoma, the receipt of chemotherapy and more lymph nodes examined.

It has been widely reported that the presence of PNI was a poor prognostic factor in colorectal cancer (9, 10, 12, 15, 22). In 2000, PNI was adopted as a negative prognostic factor by AJCC and the College of American Pathologists (CAP) has recommended categorization of PNI in the pathology reports for a decade (36, 37). We then validated the prognostic value of PNI in node-negative colon cancer, it was found that PNI (+) patients were independently associated 59.0 and 60.7% increased risk of colon cancer-specific mortality compared with PNI (−) patients in stage I and stage II colon cancer, respectively. In addition, the 5-year CSS rates of PNI (+) patents and PNI (−) patents were 93.6 and 96.2% in stage I colon cancer, 77.5 and 87.9% in stage II colon cancer, respectively. Thus, the poor prognosis of PNI (+) has been demonstrated in both stage I and II colon cancer in the current study.

Some previous studies reported the poor prognosis of PNI (+) might need to be mitigated by adjuvant chemotherapy in node-negative colon cancer, however, the predictive role of PNI (+) for the receipt of adjuvant chemotherapy is not yet established (7, 22, 23, 25, 38). Adjuvant chemotherapy was not traditionally used in stage I colon cancer, we then evaluated whether PNI was a predictive factor of response to adjuvant chemotherapy in stage II colon cancer. Kaplan–Meier analysis showed that the 5-year CSS rates of patents with and without the receipt of chemotherapy were 91.1 and 90.0% in T3 colon cancer without the presence of PNI, respectively; and the 5-year CSS rates of patents with and without the receipt of chemotherapy were 83.6 and 81.4% in T3 colon cancer with the presence of PNI, respectively. However, after adjusting for other prognostic factors, it was found that the receipt of chemotherapy was not an independent prognostic factor for CSS neither in T3 colon cancer without the presence of PNI nor in T3 colon cancer with the presence of PNI.

Kaplan–Meier analysis showed that patents with the receipt of chemotherapy were significantly associated with better CSS compared to those without the receipt of chemotherapy in T4 colon cancer without the presence of PNI (80.1% VS. 71.2% for 5-year CSS rate, P <0.0001); the receipt of chemotherapy was significantly associated with better CSS compared with those without the receipt of chemotherapy in T4 colon cancer with the presence of PNI (73.3% VS. 62.7% for 5-year CSS rate, P = 0.001). Moreover, after adjusting for other prognostic factors, it was found that the receipt of chemotherapy was independently associated with 34.0 and 36.0% decreased risk of cancer-specific mortality compared with those without the receipt of chemotherapy in T4 colon cancer without and with the presence of PNI, respectively. Therefore, adjuvant chemotherapy was found to provide a survival benefit in stage IIB colon cancer but not in stage IIA colon cancer, irrespective of presence of PNI. We then believed that the presence of PNI was not a predictive factor of response to adjuvant chemotherapy in node-negative colon cancer.

In 2016, a retrospective analysis was conducted by Dr. Cienfuegos and his colleagues (25), which identified 507 patients with stage I–II colon cancer from January 2000 and December 2012. They reported adjuvant chemotherapy could improve the prognosis in PNI (+) patients but not in PNI (−) patients. However, the sample size of this study was very small (n = 57 for PNI (+)), and the authors did not conduct subgroup analyses in stage IIA and stage IIB colon cancer patients. Then in 2019, Leijssen et al. (23) aimed to establish the predictive value of PNI in stage I to III colon cancer and included 1,222 pathological stage I to III colon cancer patients from a prospectively maintained survival and outcomes database. Consistent with our findings, their work also showed that a significant predictive response with adjuvant chemotherapy was not found in PNI (+) node-negative colon cancer.

The present research to the best of our knowledge is the first large population-based study to evaluate the predictive value of response to adjuvant chemotherapy of PNI in the subgroups of stage IIA and stage IIB colon cancer. We have demonstrated the poorer prognosis of PNI (+) in both stage I and II colon cancer. More importantly, in the present study, adjuvant chemotherapy was found to provide a survival benefit in stage IIB colon cancer but not in stage IIA colon cancer, irrespective of presence of PNI. The large sample size made it convincing to conclude that the presence of PNI was not a predictive factor of response to adjuvant chemotherapy in node-negative colon cancer.

Three limitations need to be addressed in this study. First, the retrospective design of this study was subject to its inherent limitations, and the results of the present study warranted replication in larger prospective studies. Second, in the era of individualized and precision medicine, the prognostic values of some biomarkers were widely recognized in colorectal cancer, but they were not included into our analyses due to the limitations of the database (39–43). Third, the detailed chemotherapy regimens were not available from the SEER database.

## Conclusion

We have demonstrated the poor prognosis of PNI (+) in both stage I and II colon cancer. More importantly, adjuvant chemotherapy was found to provide a survival benefit in stage IIB colon cancer but not in stage IIA colon cancer, irrespective of presence of PNI. The presence of PNI was not a predictive factor of response to adjuvant chemotherapy in node-negative colon cancer.

## Data Availability Statement

Publicly available datasets were analyzed in this study. This data can be found here: Surveillance, Epidemiology, and End Results (SEER) Program (www.seer.cancer.gov).

## Author Contributions

YL and YX were responsible for the conception and design the study. JT, ZY and WW performed the study selection, data extraction and statistical analyses. JT and JJ were responsible for the draft of the manuscript. JT and ZY contributed to a critical revision of the manuscript. All authors have read and approved the final version of the manuscript.

## Conflict of Interest

The authors declare that the research was conducted in the absence of any commercial or financial relationships that could be construed as a potential conflict of interest.
