# Supplementary material for: Perineural Invasion Is a Strong Prognostic Factor but Not a Predictive Factor of Response to Adjuvant Chemotherapy in Node-Negative Colon Cancer
Source: Front Oncol. 2021 Mar 30;11:663154. doi: 10.3389/fonc.2021.663154 (PMC8042311; doi:10.3389/fonc.2021.663154)
Supplement: Supplementary file 1 [file Table_1.docx]

**Table S1. Univariate and multivariate survival analyses of T3N0M0 colon cancer (to evaluate the efficacy of chemotherapy with the presence of PNI).**

| **Variable** | **Univariate** | |  | **Multivariate** | |
| --- | --- | --- | --- | --- | --- |
|  | **HR (95%CI)** | ***P*** |  | **HR (95%CI)** | ***P*** |
| **Age at diagnosis** |  | <0.001 |  |  | <0.001 |
| **≤65** | Reference |  |  | Reference |  |
| **>65** | 2.072 (1.863-2.306) |  |  | 2.038 (1.825-2.276) |  |
| **Race** |  | 0.001 |  |  | <0.001 |
| **White** | Reference |  |  | Reference |  |
| **Black** | 1.138 (0.997-1.299) | 0.056 |  | 1.260 (1.102-1.440) | 0.001 |
| **Other** | 0.744 (0.616-0.899) | 0.002 |  | 0.786 (0.650-0.950) | 0.013 |
| **Sex** |  | 0.292 |  |  |  |
| **Male** | Reference |  |  |  |  |
| **Female** | 1.048 (0.960-1.145) |  |  |  |  |
| **Year** |  | 0.068 |  |  | 0.204 |
| **2010** | Reference |  |  | Reference |  |
| **2011** | 0.957 (0.840-1.091) | 0.512 |  | 0.965 (0.847-1.099) | 0.591 |
| **2012** | 0.946 (0.825-1.084) | 0.424 |  | 0.958 (0.836-1.099) | 0.539 |
| **2013** | 0.871 (0.750-1.012) | 0.072 |  | 0.878 (0.755-1.020) | 0.089 |
| **2014** | 0.851 (0.721-1.003) | 0.055 |  | 0.892 (0.756-1.052) | 0.175 |
| **2015** | 0.758 (0.623-0.922) | 0.006 |  | 0.794 (0.652-0.966) | 0.021 |
| **Grade** |  | 0.192 |  |  | 0.406 |
| **I** | Reference |  |  | Reference |  |
| **II** | 1.050 (0.878-1.254) | 0.595 |  | 1.057 (0.885-1.263) | 0.542 |
| **III** | 1.210 (0.985-1.485) | 0.069 |  | 1.186 (0.965-1.457) | 0.105 |
| **IV** | 1.063 (0.773-1.462) | 0.708 |  | 1.058 (0.768-1.455) | 0.731 |
| **Unknown** | 1.220 (0.812-1.834) | 0.339 |  | 1.139 (0.758-1.713) | 0.531 |
| **Histological type** |  | 0.459 |  |  |  |
| **Adenocarcinoma** | Reference |  |  |  |  |
| **Mucinous/signet-ring cell carcinoma** | 0.944 (0.811-1.099) |  |  |  |  |
| **Total number of lymph nodes examined** |  | <0.001 |  |  | <0.001 |
| **<12** | Reference |  |  | Reference |  |
| **≥12** | 0.539 (0.482-0.604) |  |  | 0.561 (0.501-0.629) |  |
| **Perineural invasion, chemotherapy** |  | <0.001 |  |  | <0.001 |
| **None, no/unknown** | Reference |  |  | 0.568 (0.479-0.673) | <0.001 |
| **None, yes** | 0.793 (0.678-0.928) | 0.004 |  | 0.535 (0.428-0.670) | <0.001 |
| **Present, no/unknown** | 1.749 (1.476-2.073) | <0.001 |  | Reference |  |
| **Present, yes** | 1.269 (0.868-1.856) | 0.219 |  | 0.927 (0.613-1.400) | 0.717 |

**Table S2. Univariate and multivariate survival analyses of T4N0M0 colon cancer (to evaluate the efficacy of chemotherapy with the presence of PNI).**

| **Variable** | **Univariate** | |  | **Multivariate** | |
| --- | --- | --- | --- | --- | --- |
|  | **HR (95%CI)** | ***P*** |  | **HR (95%CI)** | ***P*** |
| **Age at diagnosis** |  | <0.001 |  |  | <0.001 |
| **≤65** | Reference |  |  | Reference |  |
| **>65** | 1.655 (1.437-1.908) |  |  | 1.417 (1.216-1.651) |  |
| **Race** |  | 0.153 |  |  | 0.014 |
| **White** | Reference |  |  | Reference |  |
| **Black** | 1.183 (0.971-1.440) | 0.095 |  | 1.324 (1.085-1.616) | 0.006 |
| **Other** | 0.897 (0.689-1.167) | 0.419 |  | 0.913 (0.701-1.189) | 0.500 |
| **Sex** |  | 0.003 |  |  | 0.051 |
| **Male** | Reference |  |  | Reference |  |
| **Female** | 1.223 (1.072-1.395) |  |  | 1.141 (0.999-1.303) |  |
| **Year** |  | 0.690 |  |  |  |
| **2010** | Reference |  |  |  |  |
| **2011** | 1.068 (0.873-1.308) | 0.522 |  |  |  |
| **2012** | 0.946 (0.765-1.169) | 0.606 |  |  |  |
| **2013** | 1.048 (0.841-1.307) | 0.676 |  |  |  |
| **2014** | 0.909 (0.715-1.157) | 0.439 |  |  |  |
| **2015** | 0.927 (0.707-1.216) | 0.483 |  |  |  |
| **Grade** |  | 0.001 |  |  | 0.016 |
| **I** | Reference |  |  | Reference |  |
| **II** | 0.928 (0.706-1.221) | 0.595 |  | 0.950 (0.722-1.252) | 0.717 |
| **III** | 1.289 (0.960-1.730) | 0.092 |  | 1.246 (0.925-1.678) | 0.147 |
| **IV** | 1.170 (0.788-1.736) | 0.436 |  | 1.168 (0.786-1.736) | 0.442 |
| **Unknown** | 1.097 (0.652-1.847) | 0.726 |  | 1.183 (0.702-1.993) | 0.528 |
| **Histological type** |  | 0.508 |  |  |  |
| **Adenocarcinoma** | Reference |  |  |  |  |
| **Mucinous/signet-ring cell carcinoma** | 0.938 (0.775-1.134) |  |  |  |  |
| **Total number of lymph nodes examined** |  | <0.001 |  |  | <0.001 |
| **<12** | Reference |  |  | Reference |  |
| **≥12** | 0.521 (0.443-0.612) |  |  | 0.508 (0.431-0.598) |  |
| **Perineural invasion, chemotherapy** |  | <0.001 |  |  | <0.001 |
| **None, no/unknown** | Reference |  |  | 0.686 (0.552-0.852) | 0.001 |
| **None, yes** | 0.583 (0.499-0.683) | <0.001 |  | 0.453 (0.354-0.579) | <0.001 |
| **Present, no/unknown** | 0.802 (0.577-1.114) | 0.188 |  | Reference |  |
| **Present, yes** | 1.497 (1.207-1.855) | <0.001 |  | 0.640 (0.438-0.935) | 0.021 |
